# Derivation of Rhesus Monkey Parthenogenetic Embryonic Stem Cells and Its MicroRNA Signature

**DOI:** 10.1371/journal.pone.0025052

**Published:** 2011-09-26

**Authors:** Qiang Wei, Zhenghua Sun, Xiechao He, Tao Tan, Bin Lu, Xiangyu Guo, Bing Su, Weizhi Ji

**Affiliations:** 1 Department of Reproduction and Development, Kunming Institute of Zoology, Chinese Academy of Sciences, Kunming, China; 2 State Key Laboratory of Genetic Resources and Evolution, Kunming Institute of Zoology, Chinese Academy of Sciences, Kunming, China; 3 Graduate School of Chinese Academy of Sciences, Beijing, China; Emory University, United States of America

## Abstract

Parthenogenetic embryonic stem cells are considered as a promising resource for regeneration medicine and powerful tools for developmental biology. A lot of studies have revealed that embryonic stem cells have distinct microRNA expression pattern and these microRNAs play important roles in self-renewal and pluripotency of embryonic stem cells. However, few studies concern about microRNA expression pattern in parthenogenetic embryonic stem cells, especially in non-human primate—the ideal model species for human, largely due to the limited rhesus monkey parthenogenetic embryonic stem cells (rpESCs) available and lack of systematic analysis of the basics of rpESCs. Here, we derived two novel rpESCs lines and characterized their microRNA signature by Solexa deep sequencing. These two novel rpESCs shared many properties with other primate ESCs, including expression of pluripotent markers, capacity to generate derivatives representative of all three germ layers in vivo and in vitro, maintaining of euploid karyotype even after long culture. Additionally, lack of some paternally expressed imprinted genes and identity of Single-nucleotide Polymorphism (SNP) compare to their oocyte donors support their parthenogenesis origin. By characterizing their microRNA signature, we identified 91 novel microRNAs, except those are also detected in other primate ESCs. Moreover, these two novel rpESCs display a unique microRNA signature, comparing to their biparental counterpart ESCs. Then we analyzed X chromosome status in these two novel rpESCs; results suggested that one of them possesses two active X chromosomes, the other possesses only one active X chromosome liking biparental female embryonic stem cells. Taken together, our novel rpESCs provide a new alternative to existing rhesus monkey embryonic stem cells, microRNA information expands rhesus monkey microRNA data and may help understanding microRNA roles in pluripotency and parthenogenesis.

## Introduction

Parthenogenetic activated blastocysts are promising resource for derivation of embryonic stem cells (ESCs), owing to comparable isolation efficiency with biparental counterparts and histocompatibility with oocyte donors [1]–[3]. Because of lacking paternal genome, parthenogenetic ESCs have unique imprinting pattern and have aroused a lot of interest. Rhesus monkey (*Macaca mulatta*), one of the most well-studied non-human primate, is an ideal model for ESCs-based cell replacement therapy due to its genetic and physiological similarity with human [4], [5]. However, there are only limited studies [6], [7] concerned about the rhesus monkey parthenogenetic embryonic stem cells (rpESCs), largely due to the limited rpESCs lines available and lack of systematic analysis of the basics of rpESCs.

MicroRNA (miRNA) belong to a group of single-stranded noncoding RNAs that are 21–23 nucleotides (nt) in length [8]. They bind to the complementary sites within the 3′UTRs of cognate mRNAs, leading to the degradation, deadenylation, or translational repression of the target mRNAs, which provide a crucial and pervasive post-transcriptional gene regulation [9]. Increasing evidence suggests that miRNAs play important role in self-renewal, differentiation and reprogramming of ESCs or iPS [10], [11]. Investigations of the miRNA expression patterns in human ESCs have revealed a unique miRNA signature distinct from differentiated cells [12]–[18]. Our previous research suggested that rhesus monkey ESCs from in vitro fertilization (IVF) blastocysts also share a unique miRNAs set, miR-302 cluster, which were reported to be highly enriched in human ESCs, were the most rhesus monkey ESCs enriched miRNAs [19]. The parthenogenetic ESCs would exhibit many themselves own characteristics, due to the absence of paternal genome, although they share many properties with their biparental counterparts. However, the miRNA expression profiling in parthenogenetic ESCs is largely unknown.

In current study, we isolated two novel rpESCs, and characterized their miRNA signature by a novel, robust and reproducible Solexa deep sequencing approach. On basis of sequencing data, we compared rpESCs with IVF rhesus monkey ESCs, and analyzed their X chromosome status.

## Results

### Isolation and characterization of two novel rhesus monkey parthenogenetic ESCs

**Figure 1:**
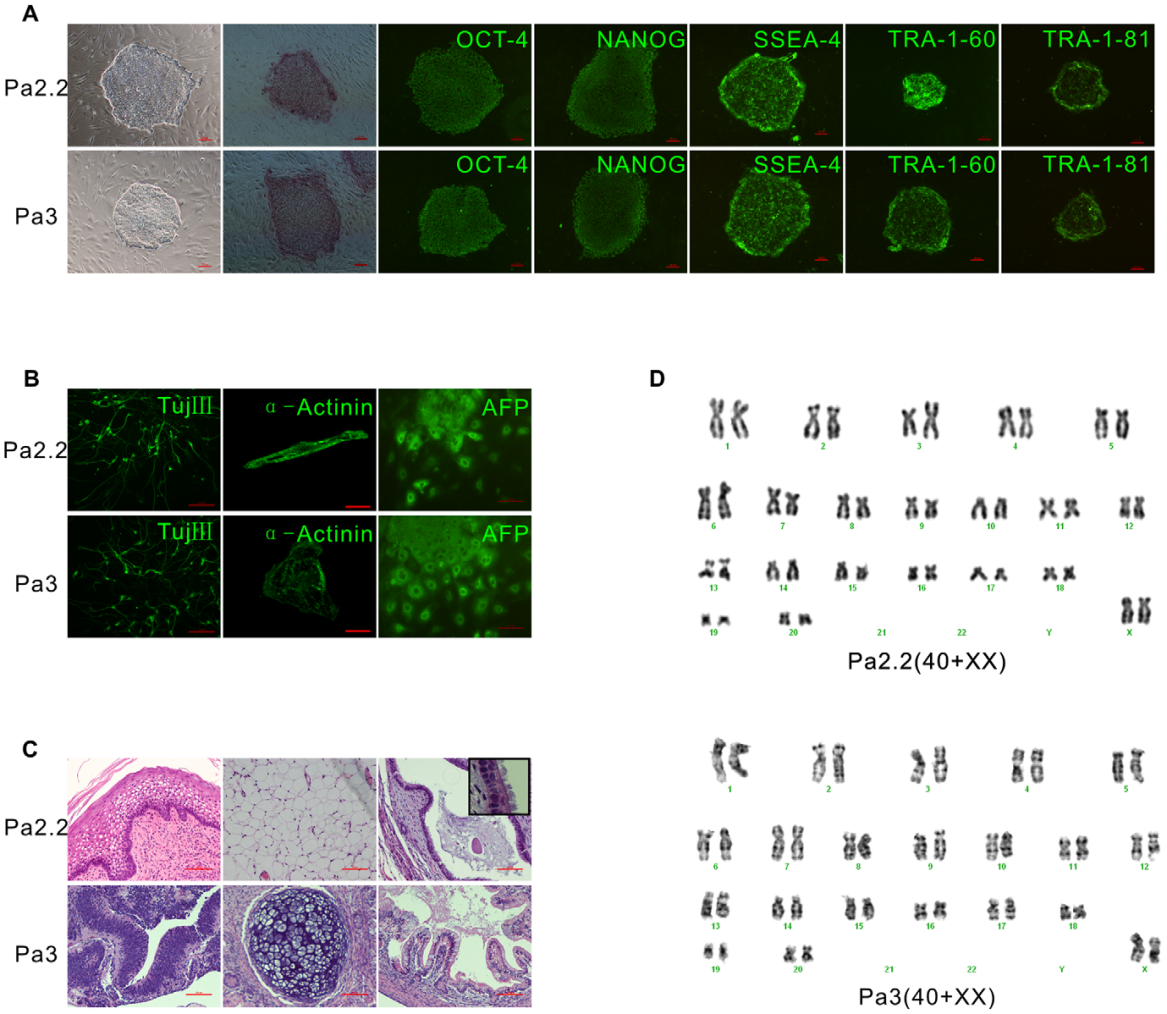
Isolation and characterization of two novel rhesus monkey parthenogenetic ESCs. A, morphology and pluripotent markers. From left to right are Phase-contrast micrograph of an ESC colony growing on mEFs, Alkaline phosphatase staining, OCT-4, NANOG, SSEA-4, TRA-1-60 and TRA-1-81. Up is Pa2.2, down is Pa3; B, differentiation in vitro. From left to right are neuronal marker Tuj III, Cardiomyocytes marker α-cardiac actinin and Endoderm marker α-fetoprotein. Up is Pa2.2, down is Pa3; C, teratomas. Up is Pa2.2, from left to right are Squamous epithelium, adipose and respiratory epithelia (inset shows respiratory cilia). Down is Pa3, from left to right are neural tube, cartilage and intestinal epithelia; D, G-banding karyotypes. Up is Pa2.2, down is Pa3; Size bar (A, 100 µm; B and C, 50 µm).

A total of 7 parthenogenetic activated blastocysts with prominent inner cell mass(ICM) were selected for immunosurgery, and 3(42%) ICMs were successfully isolated and plated on feeder cells. All the ICMs attached to the feeder cells within 48 hours, and 2(28%) ESC-like ICM-outgrowths appeared after 7∼8 days. These ICM-outgrowths were manually dissociated into 4∼6 smaller clumps with a microscalpel, excised from the feeder cells and replated onto fresh feeder cells. Clones with distinct boundary and a high nuclear to cytoplasmic ratio were selected for further propagation. Finally, two rhesus monkey parthenogenetic ESC lines were obtained (28%), designated as Pa2.2 and Pa3. These two novel rpESCs shared common morphological property with other primate ESCs: flat clones, remarkable boundary with feeder cells, high ratio of nuclear to cytoplasmic and prominent nucleoli (Fig. 1A). These two novel rpESCs showed strong alkaline phosphatase activity (Fig. 1A), and expressed high level pluripotent markers, including Oct-4, Nanog, SSEA-4, TRA-1-60, TRA-1-81 (Fig. 1A), Sox-2 and Rex-1 (Fig S1).

These two rpESCs could spontaneously differentiate into cell lineages representative of all three embryonic germ layers by embryoid body (EB) formation method, including neuron-like cells, contractive cardiomyocytes and endoderm-like cells (Fig. 1B).

When the rpESCs clones were injected into the hind leg muscle of SCID mice, after 6∼7 weeks, teratomas could be detected, and the histological characterization revealed that ectoderm-(neural tube and Squamous epithelium), mesoderm-(cartilage, adipose), and endoderm-(intestinal and respiratory epithelia) like structures were present in teratomas from these two novel rpESCs (Fig. 1C).

Pa2.2 have been cultured for more than 120 passages, and Pa3 for more than 80 passages. Detailed G-banding analysis revealed that all of the two rESC lines were karyotypically normal, with a diploid set of 42 chromosomes (40+XX) (Fig. 1D), even after long time cultivation in vitro and repeat freezing.

### Identification of parthenogenetic origin

**Figure 2:**
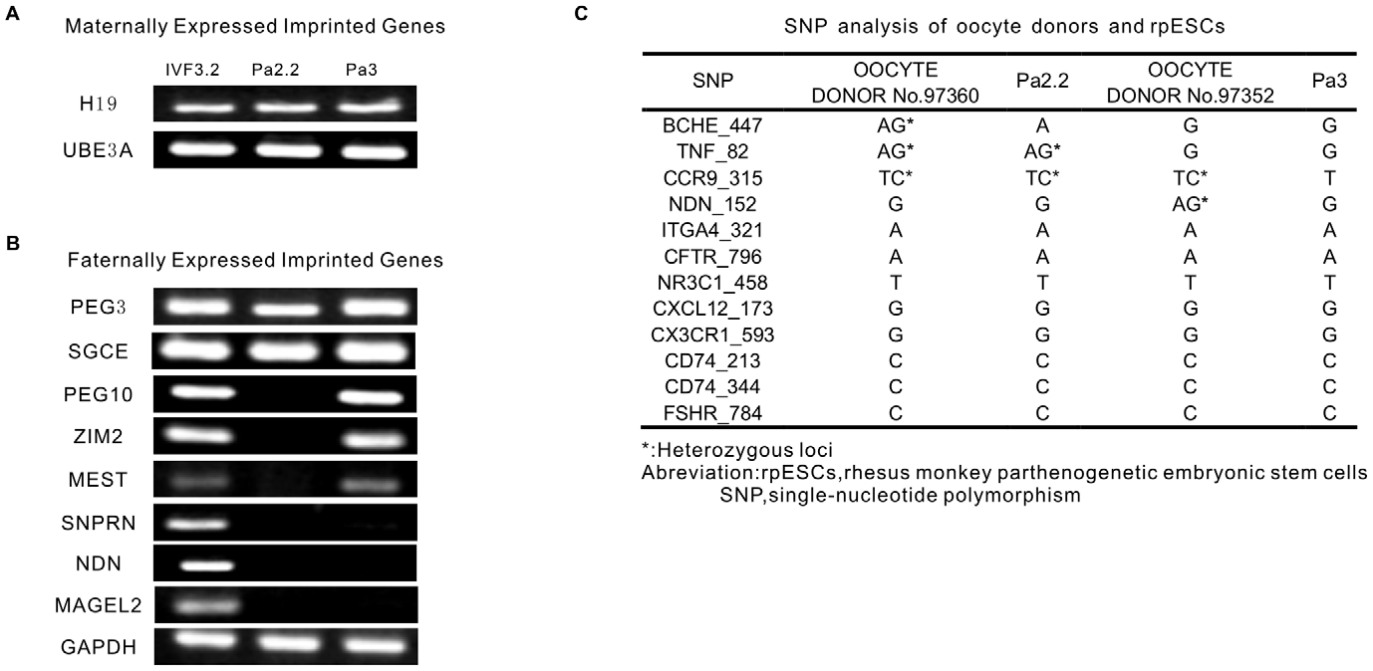
Expression of maternally and paternally expressed imprinted genes. A, expression of maternally expressed imprinted genes in IVF3.2 (female IVF rhesus monkey ESCs) and rpESCs. B, Expression of paternally expressed imprinted genes in IVF3.2 (female IVF rhesus monkey ESCs) and rpESCs. C, SNP analysis of oocyte donors and rpESCs.

In normal fertilized embryonic stem cells, imprinted genes express predominantly from maternal or paternal alleles respectively. Paternally expressed imprinted genes would not be detectable in rpESCs, since both alleles are of maternal origin. We used RT-PCR to analysis two maternally and eight paternally expressed imprinted genes in our novel rpESCs and a IVF-origin rhesus monkey ESCs control (IVF3.2) [19]. Expectably, H19 and UBE3A, two imprinted genes normally expressed predominantly from the maternal allele were detected in both rpESCs and IVF-origin control (Fig. 2A). However, paternally expressed imprinted genes, PEG3 and SGCE were also detected in rpESCs (Fig. 2B). In addition, paternally expressed imprinted genes including PEG10, ZIM2 and MEST were expressed in Pa3, but not in Pa2.2 (Fig. 2B). Only three paternally expressed imprinted genes we tested, SNPRN, NDN and MAGEL2, were absent from all two rpESCs (Fig. 2B). All of these eight paternally expressed imprinted genes could be detected in IVF–origin control (Fig. 2B).

Since the diploid genome in parthenogenetic ESCs were origined from identical sister chromatids in MII oocyte by retention of the second polar body, on the hand, single-nucleotide polymorphism (SNP) would be identical between oocyte donors and their daughter cells, on the other hand, each homologous chromosome pair in parthenogenetic ESCs might be expected to display high levels of homozygosity. Indeed, twelve SNP loci we analyzed were uniform between Pa2.2 and its oocyte donor 97360# monkey, Pa3 and its oocyte donor 97352# monkey respectively (Fig. 2C). However, two of twelve SNP loci we analyzed, TNF-82 and CCR9-325, displayed heterozygosity in Pa2.2 rpESCs, but Pa3 rpESCs displayed homozygosity in all of these twelve SNP loci (Fig. 2C).

Although some paternally expressed imprinted genes indeed were detectable in two rpESCs, lack of other paternally expressed imprinted genes and identity of SNP between rESCs and oocyte donors could be as evidence for parthenogenetic origin.

### Small RNA Sequencing and miRNA expression profiles in the rpESCs

**Figure 3:**
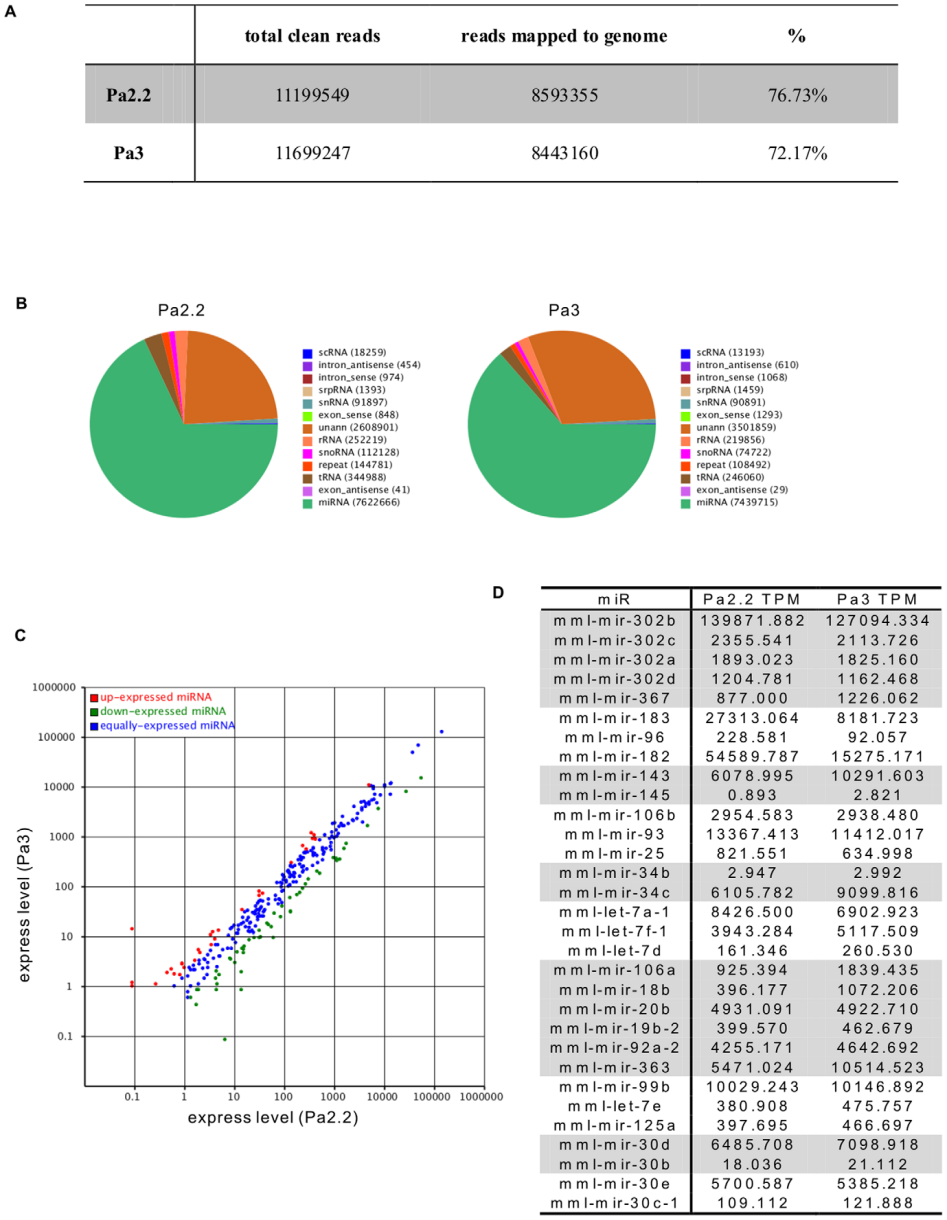
MiRNA expression profiles in the rpESCs. A, the counts and proportion of the mapped tags in the total clean reads; B, annotation of 13 RNA categories in the pie chart for Pa2.2 and Pa3 respectively. C, Scatter Plot of the expression of kwon miRNA in two novel rpESCs. D, rpESCs enriched miRNAs clusters, the expression of miRNA were normalized as transcript per million(TPM). MiRNAs were considered to belong to the same genomic cluster if the genomic locations of the first nucleotides of the predicted pre-miRNA hairpins were within 10 kilobases (kb).

Sequencing of small RNA libraries yielded 12861523 and 12922804 raw reads from Pa2.2 and Pa3 respectively. After filtering, we obtained 11199549 (Pa2.2) and 11699247 (Pa3) clean reads (18–30 nt). The length distribution of these clean reads centers at 22–23 nt, coinciding with the scope of miRNA length. Aligned with the rhesus monkey genome (rheMac2) by SOAP (Short Oligonucleotide Alignment Program) [20], a total of 8593355 (Pa2.2) and 8443160 (Pa3) sequences were mapped to the genome (Fig. 3A). It showed that miRNA was the major component in the small RNA library of rpESCs (Fig. 3B), about 60% of which were matched to reference miRNAs in each sample, and the proportion of other annotated RNAs was less than 15%. Notably, about 25% of the sequences could not be annotated because of the low coverage (6×) and relatively poor annotations of the released rhesus macaque genome. These unknown sequences were subjected to further analysis for identifying novel miRNAs.

After matched to 466 reference miRNAs of rhesus macaque, 345and 346 known miRNAs were detected inPa2.2 and Pa3, respectively, with absolute counts ranging from 1 to 1566502 (Table S2). The normalized expression of miRNA was reflected by the expression of transcript per million (TPM). Comparison of the known miRNA between Pa2.2 and Pa3 showed that the holistic expression profiles in these two novel parthenogenetic rESCs were similar, because most of the miRNAs were expressed consistently (Fig. 3C).

We also analyzed miRNA cluster expression patterns and the results revealed that miRNAs from miR-302 cluster, miR-182/miR-183/miR-96 cluster and miR-143/miR-145 cluster were the most rpESCs enriched miRNAs. Fig. 3D gave the top 10 enriched miRNAs clusters in rpESCs. Interestingly, miR-371–373 cluster, which was reported to be a highly enriched miRNA cluster in human ESCs [12], [14], [17], [18], only represented a moderate expression level in our in rpESCs (table S3). In addition, miRNAs from C19MC, another human ESCs-enriched miRNAs cluster, was lower than minimum threshold expression level in rpESCs (table S3).

### Identification of novel miRNAs

We predicted novel miRNA genes among the unannotated sequences by Mireap (http://sourceforge.net/projects/mireap/). Briefly, according to the characteristic hairpin structure of miRNA precursor, the unannotated small RNA tags, which could be mapped to genome, were downloaded with their flanking sequences and analyzed for secondary structure, Dicer cleavage site and minimum free energy (mfe). Eventually, there were 91 novel miRNAs shared by these two novel rpESCs (table S4), and 23 of them had the homologs to reference miRNAs (miRBase V16.0) in other species.

### Comparison of miRNA expression between parthenogenetic and IVF rhesus monkey ESCs

**Figure 4:**
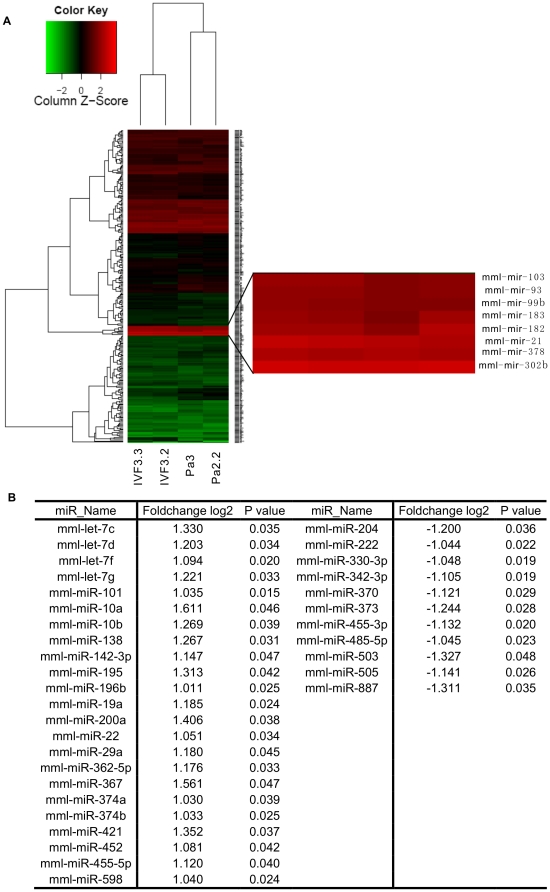
Comparison of miRNA expression between parthenogenetic and IVF rhesus monkey ESCs. A, heat-map of hierarchical clustering of miRNAs expression in parthenogenetic and IVF rhesus monkey ESCs. Right shows miRNAs whose expression more than 10000 counts TPM. B, miRNAs remarkable up- or down-regulated(fold-change>2,p<0.05) in rpESCs comparing with IVF rhesus monkey ESCs.

Although parthenogenetic ESCs shared many properties with their IVF counterpart, the question are there some difference in miRNA level between them is still unknown. Here, we compared miRNA expression profiles of 230 miRNAs(counts TPM>3) in rpESCs with IVF-origin rhesus monkey ESCs, IVF3.2 and IVF3.3, which we recently reported [19]. With respect to comprehensive miRNAs expression pattern, both of two type rhesus monkey ESCs represented high similarity (Fig. 4A). Especially, all of miR-302b, miR-182, miR-183, miR-93 and miR-99b, which were from rpESCs enriched miRNAs cluster (Fig. 3D), were also highly expressed in IVF3.2 and IVF3.3 (counts TPM>10000). Additionally, miR-103, miR-21 and miR-378 possessed very high expression level (counts TPM>10000) in despite of origin from parthenogenesis or IVF. Whereas, hierarchical clustering analysis distinctly separated rpESCs from IVF-origin rhesus monkey ESCs (Fig. 4A), indicating that rpESCs had a distinguishable signature of miRNA expression. Therefore, we listed remarkable up- or down-regulated(fold-change>2,p<0.05) miRNAs comparing with IVF-origin rhesus monkey ESCs (Fig. 4B).

### X-chromosome status in rpESCs

Since both X-chromosome in parthenogenetic ESCs were origined from single X-chromosome in MII oocyte, epigenetic status of X-chromosome, especially for X-chromosome inactivation (XCI), were very important for parthenogenetic ESCs. Therefore, we analysed X-chromosome status in our two novel rpESCs based on miRNA expression data.

**Figure 5:**
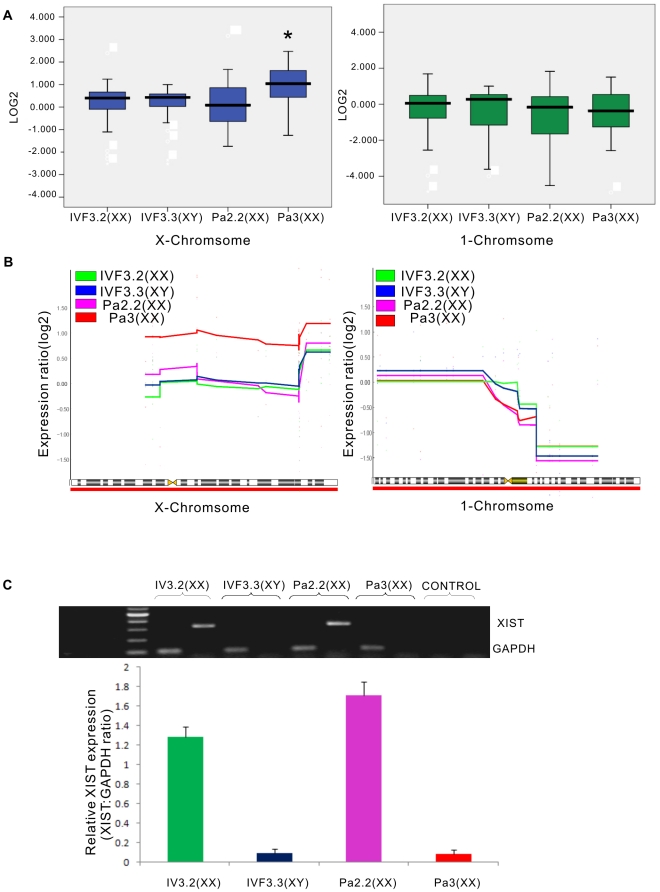
Analysis of X-chromosome status in rpESCs based on miRNAs expression. A, box plot of the entire set of kwon miRNAs on X or 1 chromosome. Left, X-chromosome, right, 1-chromosome. (*, p<0.05). B, moving average plot of the X or 1 chromosome. Shown are relative gene expression levels along the X or 1 chromosome. Left, X-chromosome, right, 1-chromosome. IVF3.2 rhesus monkey ESCs(female,green), IVF3.3 rhesus monkey ESCs (male, blue), Pa2.2 rpESCs (mauve) and Pa3 rpESCs(red). C, analysis of XIST expression by semi- quantitive PCR. Histogram showed relative XIST expression in IVF3.2 rhesus monkey ESCs (female, green), IVF3.3 rhesus monkey ESCs (male, blue), Pa2.2 rpESCs(mauve) and Pa3 rpESCs (red).The relative XIST expression was XIST∶GAPDH ratio.

For total expression pattern of chromosome-linked miRNAs, we observed that X-linked miRNAs were obviously overexpressed in Pa3 (p<0.05) comparing with Pa2.2 and other two IVF-origin rhesus monkey ESCs (one female IVF3.2, one male IVF3.3)(Fig. 5A Left). However, Pa2.2 and two IVF-origin rhesus monkey ESCs had no statistical difference (Fig. 5A left). Simultaneously, the expression level of 1-chromosome-linked miRNAs was similar in all rhesus monkey ESCs (p>0.05) (Fig. 5A right). Furthermore, we visualized expression profiles of chromosome-linked miRNAs using CGH-Explorer. Similar to the total expression pattern, in moving average plot of the X chromosome, X-linked miRNAs in Pa3 were prominently upregulated compared with Pa2.2, IVF3.2 and IVF3.3, although all of rhesus monkey ESCs had an accordant trend (Fig. 5B left). Whereas, the other rpESC, Pa2.2 was consist with IVF3.2 and IVF3.3 (Fig. 5B left). For expression profiles of 1-chromosome-linked miRNAs, no matter of origin from parthenogenesis or IVF, all of rhesus monkey ESCs showed a very parallel moving average plot (Fig. 5B right). These results suggested that both of X chromosomes were activated in Pa3, while one of them was inactivated in Pa2.2.

In order to further validate the results detailed above, we measured XIST, which was expressed in XCI, using semi-quantitive PCR. Expectably, the transcript of XIST had a high expression level in IVF3.2 and Pa2.2. On the other hand, the RT-PCR products of XIST could not be detected in IVF3.3 and Pa3 (Fig. 5C). These results supported the coclusion above that Pa3 had two activated X chromosomes, but Pa2.2 had only one.

## Discussion

Here, we isolated two novel rpESCs on 28% efficiency, comparable to biparental counterpart. These rpESCs shared many properties with other primate ESCs, including expression of pluripotent markers, capacity to generate derivatives representative of all three germ layers in vivo and in vitro, maintaining of euploid karyotype even after long culture. Because of lack of paternal genome, paternally expressed imprinted genes were expected to be absence in rpESCs. However, in our two novel rpESCs, paternally expressed imprinted genes including PEG3, SGCE, PEG10, ZIM2 and MEST could be detectable at least in one rpESCs, consist with other available parthenogenetic rhesus monkey ESCs [7]. Nevertheless, SNPRN, NDN and MAGEL2 were absent from both our rpESCs and others [7]. Although twelve SNP loci we analyzed were uniform between rpESCs and their oocyte donors, Pa2.2 represented heterozygousity to some extent. This phenomenon recently observed also in mouse and human parthenogenetic ESCs [1], [21], [22], due to homologous crossing-over and recombination during meiosis I.

In our two rpESCs, mir-302 cluster had a very high expression level, consisting with previous studies that mir-302 cluster was high-riched in human ESCs [12]–[18]. Intriguingly, mir-302 cluster can reprogram cancer or somatic cells into iPS [23]–[25] revealed that mir-302 cluster had important roles in maintaining pluripotency of pluripotent cells. Recently, the roles of mir-302 cluster in regulation of ESCs pluripotency have been clarified on some extent. Nanog, Oct3/4, Sox2 and Rex1 are upstream regulators of the miR-302 cluster promoter [26], miR-302a regulate ESCs cell cycle by repressing cyclin D1 [27], and miR-302 impair early differentiation by repress NR2F2 [28] and Lefty [29] at the post-transcriptional level. On the other hand, miR-302 can enhance reprogramming by accelerating mesenchymal-to-epithelial translation (MET) [30] and suppressing some epigenetic regulators [23]. MiR-106a-363 cluster and miR-106b-25 cluster, which were also high-riched in rpESCs, were reported to enhance reprogramming by accelerating MET [30], [31]. Interestingly, miR-371–373 cluster and C19MC miRNAs, which were reported to be highly enriched in human ESCs [14], [18], only represented a moderate or almost undetectable expression level in our rpESCs respectively. Additionally, some members of let-7 family were upregulated in rpESCs. These results were also observed in some human ESCs lines [13], [16]. This variance in some miRNAs may reflect difference in culture condition, developmental stage when ICM were isolated.

Although rpESCs shared many properties with their IVF counterpart, comparing between them on miRNA level would help understanding universality and particularity of rpESCs. For miRNAs which had a very high expression level(>10000 reads TPM) in both parthenogenetic and IVF rhesus monkey ESCs, members of mir-302 cluster, miR-106b-25 cluster have important roles in maintaining of pluripotency. Interestingly, Guo and colleagues recently reported [32] that members of miR-99b-125a cluster increased the number of hematopoietic stem cells in vivo by inhibiting pro-apoptotic genes Bak1. This cluster was also up-regulated expression in our rhesus monkey ESCs despite of origin from parthenogenesis or IVF, suggesting the potential roles of this cluster in ESCs and IPS. Consisting with Laurent and colleagues' reports [14], miR-183–182 cluster, miR-103, miR-21 and miR-378, which were reported to be related to many type carcinomas [33]–[36], were also enriched in both types of rhesus monkey ESCs. Our results revealed that rpESCs and IVF-origin rhesus monkey ESCs represented high similarity with respect to comprehensive miRNA expression pattern, especially for miRNAs which involved in properties of stem cells. Since parthenogenetic ESCs were considered as potential source for regeneration medicine, their tumorigenesis risk should be comprehensively and carefully evaluated. By comparing with IVF-origin rhesus monkey ESCs, our results indicated that rpESCs would up- or down-regulated some specific tumor-related miRNAs, in addition to up-regulating of some oncogenic miRNAs in both type of rhesus monkey ESCs mentioned above. Oncogenic miRNAs [37], [38], including let-7 family, miR-374 family, miR-138, miR-195, miR-19a and miR-200a, had more than twice expression level in rpESCs comparing with their IVF counterparts. Reversely, tumor suppressor, including miR-222, miR-342-3p, were remarkably down-regulated in rpESCs. These results, along with recent report that cell lines from human parthenogenetic embryos had aberrant centriole distribution [39], highlighted the tumorigenesis risk of parthenogenetic ESCs.

X-chromosome inactivation, by which dosage compensation of the sex chromosome is achieved, is widely presented in mammals [40]. In mouse embryonic stem cells (mESCs) and mouse-induced pluripotent stem cells (miPSCs) both X chromosomes are active, and inactivation occurs during differentiation [41]–[43]. The presence of two active X chromosomes have been considered as one of epigenetic markers for murine ESCs. However, in human ESCs or iPS, X chromosomes status represented complex variations [44]–[46]. Especially, by changing culture and growth factor condition, pre-X inactivation human ESCs or iPS could be derived [47], [48]. By collecting and analysing the expression of the entire set of genes on the X chromosome, Bruck and colleagues divided human pluripotent cell lines into three categories: no XCI, full XCI, and partial XCI [49]. In current study, we collected and analyzed the expression of the entire set of known miRNAs on the X chromosome in our rhesus monkey ESCs. The X-linked miRNAs in Pa3 rpESCs had about twice level of expression than that of Pa2.2 rpESCs and their IVF counterparts, implying the activation of both two of X chromosome in Pa3. In order to validate this prediction, we checked XIST, a non-code RNA which was specifically expressed in female cells and took part in XCI. Expectably, XIST could not be detected in Pa3 rpESCs and male rhesus monkey ESCs IVF3.3. Whereas, Pa2.2 expressed high level of XIST, equal to female rhesus monkey ESCs IVF3.2. These results suggested that the variations of X chromosome status were also presented in parthenogenetic ESCs, regardless of the identical origin of both X chromosome. Notably, in Rhesus monkey, both X chromosomes are active in blastocyst stage from which ESCs were isolated [50]. Therefore, XCI would occur during derivation of ESCs.

## Materials and Methods

### Ethics Statement

Mature rhesus monkey were supplied by Kunming Primate Research Center, and housed in individual cages when used for the present study. This study was carried out in strict accordance with the recommendations of the Weatherall report, “The use of non-human primates in research”. The protocols involving rhesus monkey and mouse were approved by the Institutional Animal Care and Use Committee(s)(IACUC) of the Kunming Primate Research Center, the Chinese Academy of Sciences(approval ID: 20091007). All surgery was performed under sodium pentobarbital anesthesia, and all efforts were made to minimize suffering.

### Collection of Rhesus Monkey Oocytes,Parthenogenetic Activation and Embryo Culture

Cycling females were subjected to follicular stimulation using twice-daily intramuscular injections of 18 IU of recombinant human FSH (rhFSH) (Gonal FTM Laboratories) for 8 d; then 1,000 IU of human chorionic gonadotropin (hCG) (Lizhu Groups) were injected on day 9 as described by Yang et al [51]. Cumulus–oocyte complexes were collected from animals by laparoscopic follicular aspiration 30–34 h following hCG administration. Follicular contents were placed in Hepesbuffered Tyrode's albumin lactate pyruvate (TALP) medium [52] containing 0.3% BSA at 37°C. Oocytes were stripped of cumulus cells by pipetting after brief exposure (<1 min) to hyaluronidase (0.5 mg/mL) in TALP-Hepes to allow visual classification of nuclear maturity as prophase I (PI; intact germinal vesicle), metaphase I (MI; no germinal vesicle, no polar body), metaphase II (MII; first polar body present), and atretic (presence of fragmentation or vacuoles in ooplasm). Immature oocytes in either MI or PI stages were cultured in a 50-µL drop of CMRL-1066 medium (Invitrogen) containing 10% FBS, 10 IU/mL porcine FSH, and 10 IU/mL ovine luteinizing hormone at 37°C in humidified air (5% CO_2_) for up to 24 h. Oocytes that were mature (MII) at collection were placed in chemically defined, protein-free hamster embryo culture medium-10 (HECM-10) [53] at 37°C in 5% CO_2_ until parthenogenetic activation. Mature (MII) oocytes were parthenogenetic activated by electroporation with two 40-µsec direct current pulses of 200 V/mm (BTX ECM-2000; BTX, Inc.,San Diego, CA) in 0.25 M D-Mannitol buffer containing 0.05 mM calcium chloride, 0.1 mM magnesium chloride, 0.5 mM Hepes, and 0.02% fatty acid-free BSA. Electroporated oocytes were exposure to 5 µM ionomycin (Calbiochem, La Jolla, CA) for 4 min in TALP/Hepes medium supplemented with 1 mg/ml BSA and then washed for 5 min in TALP/Hepes supplemented with 30 mg/ml BSA; ionomycin-treated oocytes were then transferred into CMRL-1066 medium containing 2 mM 6-dimethylaminop urine (6-DMAP) and cultured at 37°C in 5% CO_2_ balance air for 5 h. After activation, oocytes were cultured in HECM-9 containing 10% FBS (HyClone Laboratories Inc.) to allow embryo development. Culture medium was replaced every other day.

### Embryonic Stem Cells Derivation and Culture

Full expanded blastocysts with distinct ICM were selected to derivation of embryonic stem cells. Zonae pellucidae were removed by brief exposure to 0.5% pronase, and ICM were isolated using immunosurgery. Isolated ICM were plated on Nunc four-well dishes containing mitotically inactivated mouse embryonic fibroblasts (mEFs) and cultured in Dulbecco's Modified Eagle Medium: Nutrient Mixture F-12 (DMEM/F12,Invitrogen)medium containing 20% KO-SR (Invitrogen) supplemented with 10 ng/ml bFGF(Chemicon), 1% nonessential amino acids (Invitrogen), 0.1 mM β-mercaptoethanol(Invitrogen), 1% penicillin-streptomycin-l-glutamate(PSG)(Invitrogen). ICM that attached to the mEFs and initiated outgrowth were manually dissociated into small cell clumps with a microscalpel and replated onto new mEFs. After the first passage, colonies with ESC-like morphology were selected for further propagation, characterization and freezing. When all colonies were uniform ESC-like morphology, culture medium were replaced by DMEM/F12 medium containing 20% KO-SR supplemented with 5 ng/ml bFGF (Chemicon), 1% nonessential amino acids, 0.1 mM β-mercaptoethanol, 1% PSG. The medium was changed daily, and ESC colonies were split every 4–6 days manually or by disaggregation in collagenase IV (1 mg/ml, at 37°C for 15 minutes; Invitrogen) and replating onto dishes with fresh feeder layers. Cultures were maintained at 37°C, in 5% CO_2_.

### In vitro Differentiation of ESCs

For embryoid body (EB) formation, entire ESCs colonies were detached from feeder cells by exposure to collagenase IV (1 mg/ml, at 37°C for 15 minutes) and transferred into agar-coated dishes and cultured in suspension in Dulbecco.s modified Eagle.s medium (DMEM, high glucose, without sodium pyruvate; Gibco)containing 20% FBS, 1% nonessential amino acids, 0.1 mM β-mercaptoethanol, 1% PSG. After 5–7days, EBs were transferred into gelatin-coated dishes allowing attachment for further differentiation. Medium was changed every other day.

### Teratoma Formation

Entire ESCs colonies were detached from feeder cells by exposure to collagenase IV (1 mg/ml, at 37°C for 15 minutes) and about 2 million undifferentiated ESCs from each cell line were harvested and injected into the hind leg muscle into 4-week-old SCID, male mice using an 18-gauge needle. 6–7 weeks after injection, mice were sacrificed, and teratomas were dissected, sectioned, and histologically characterized for the presence of representative tissues of all three germ layers.

### Immunohistochemical Staining

Cells were fixed with 4% paraformaldehyde in PBS for 20 minutes at room temperature, and permeabilized in 0.1%TritonX-100 in PBS for 10 minutes at room temperature. After blocked with 3% BSA, cells were stained with primary antibodies. Cells were then rinsed three times with PBS and incubated for 60 minutes with fluorescein isothiocyanate (FITC)-conjugated secondary antibody (Santa Cruz Biotechnology). Negative controls for each fluorophore- conjugated secondary antibody were carried out without the addition of the primary antibody, and nonspecific binding of secondary antibodies was detected.

### Reverse Transcription-Polymerase Chain Reaction

Total RNA was extracted using a TRIZOL RNA isolation kit (Invitrogen Corporation) according to the manufacturer's instructions. Potential contamination from genomic DNA was eliminated by DNase digestion. Cytoplasmic RNA was reverse-transcribed to single-stranded cDNA. Aliquots of cDNA were used as a template for polymerase chain reaction (PCR) amplification with individual primer pairs for specific genes. The sense and antisense primer sequences, corresponding PCR condition, and product sizes were shown in Table S1. 5 µl of PCR products were separated on a 1.5% agarose gel and visualized by ethidium bromide staining.

### Cytogenetic Analysis

Mitotically active ESCs in log phase were incubated with 120 ng/ml colcemid for 90 minutes at 37°C in 5% CO2. Entire ESCs colonies were detached from feeder cells by exposure to collagenase IV (1 mg/ml, at 37°C for 15 minutes), treated with 0.05% trypsin at 37°C for 2 minutes and centrifuged at 200 g for 5 minutes. The cell pellet was gently resuspended in 0.075 M KCl and incubated for 20 minutes at 37°C followed by fixation with methanol/glacial acetic acid (3∶1). Fixed cells were dropped on wet slides, air dried, and baked at 90°C for 1 hour. G-banding was performed as described previously [54].

### Alkaline Phosphatase Staining

Alkaline Phosphatase staining was performed using Alkaline Phosphatase Detection Kit(Catalog number SCR004, Millipore).

### Single-nucleotide Polymorphism (SNP) Analysis

Total genomic DNA was extracted using the DNeasy Tissue Kit (Qiagen) and SNP analysis was performed using polymerase chain reaction-sequence specific primer(PCR-SSP).

### RNA Extraction and Small RNA Sequencing

Total RNA was extracted using the mirVana miRNA isolation kit (Ambion). According to the manufacturer's instructions (Illumina), small RNA fraction between 18–30 nt was isolated from total RNA by PAGE purification and ligated with a pair of adaptors to their 5′ and 3′ ends. Then the small RNA were converted to cDNA by RT-PCR and amplified using the adaptor primers. The purified DNA was used directly for cluster generation and sequencing analysis by the Illumina Genome Analyzer. These data have been deposited in the GEO database (GSE30510).

### Small RNA Annotation and Novel miRNAs Prediction

To obtain clean reads, the original 35 nt reads from sequencing were filtered by trimming the 3′ adaptors, getting rid of contaminant, too-small (<18 nt) and low-quality reads. The resulting unique sequences with associated read counts were taken as sequence tags. The clean reads were aligned with Genbank and Rfam 9.1database using BLASTn, for removing non-miRNAs(rRNA, tRNA, etc). For detecting the known miRNAs, the clean reads were matched to the reference miRNA precursor sequences of rhesus macaque (miRBase version16.0) using BLASTn software, with the parameter Expect value at 0.01. For identifying repeat associated RNA and degradation tags of mRNA, the clean reads were overlapped to repeat sequences, exons and introns of mRNAs. In the initial alignment and annotation, some small RNA tags were mapped to more than one category. To obtain uniquely mapped reads, we applied the following priority rules: rRNAetc (in which Genbank > Rfam) > known miRNA > repeat > exon > intron. The unannotated small RNA tags are named as ‘unann’. We used Mireap (http://sourceforge.net/projects/mireap/) to predict novel miRNAs.

### Differential Expression of Known miRNA

Normalization of the expression of miRNA was conducted in order to get the expression of transcript per million (TPM). Normalization formula: Normalized expression = Actual miRNA count/Total count of clean reads×1000000. Based on the randomly selected 210,000 bins (10,000 bins multiply 21 chromosomes) with each bin spanning 50 bp window, we filtered out miRNAs with sporadic reads which may be generated from potential noise, and miRNAs with normalized reads no less than 3 TPM (p-value <0.0001 based on Poisson distribution simulation) were treated as true reads for further analyses. MiRNAs were considered to belong to the same genomic cluster if the genomic locations of the first nucleotides of the predicted pre-miRNA hairpins were within 10 kilobases (kb).The fold-change of the expression of miRNA between two samples were showed as Log2-ratio.

### X-linked miRNAs Analysis

We collected X-linked miRNAs in rhesus monkey ESCs expression data from this study and our prevenient report [19] In order to making a reference baseline, the median of normalized counts of each X-linked miRNAs in male rESCs was used. Then each sample were divided by this median to obtain relative gene expression levels, and were represented as log2 ratio. For visualization of these fold-change values on X chromosome, a freely available comparative genomic hybridization (CGH) analysis software program, CGH-Explorer [55] was used. Moving average plot of X chromosomes was drawn using the moving average fit tool. On the other hand, we simultaneously used 1-chromosome as control.

## Supporting Information

Figure S1 **The PCR result for ESCs-crucial transcriptional factors.**
(TIF)

Table S1 **PCR primers and condition for gene analysis.**
(DOC)

Table S2 **Absolute counts of known miRNAs in Pa2.2 and Pa3.**
(XLS)

Table S3 **The expression of members of miR-371–373 and C19MC cluster in parthenogenetic rESCs.**
(DOC)

Table S4 **Novel miRNAs in Pa2.2 and Pa3.**
(XLS)
