# Supplementary figures and images for: Derivation of Rhesus Monkey Parthenogenetic Embryonic Stem Cells and Its MicroRNA Signature
Source: PLoS One. 2011 Sep 26;6(9):e25052. doi: 10.1371/journal.pone.0025052 (PMC3180378; doi:10.1371/journal.pone.0025052)

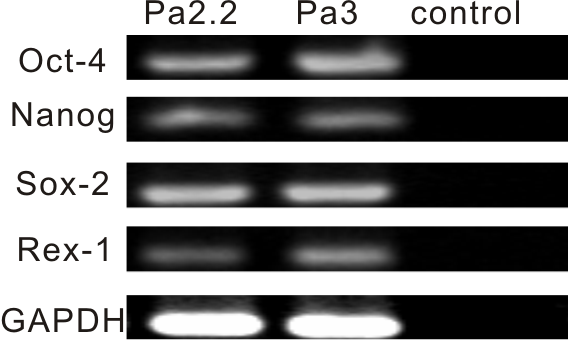

Supplement: Figure S1 — The PCR result for ESCs-crucial transcriptional factors. (TIF) [file pone.0025052.s001.tif]
