# Supplementary material for: Derivation of Rhesus Monkey Parthenogenetic Embryonic Stem Cells and Its MicroRNA Signature
Source: PLoS One. 2011 Sep 26;6(9):e25052. doi: 10.1371/journal.pone.0025052 (PMC3180378; doi:10.1371/journal.pone.0025052)
Supplement: Table S1 — PCR primers and condition for gene analysis. (DOC) [file pone.0025052.s002.doc]

**Supplemental Table 1. PCR primers and condition for gene analysis**

| Genes | Primer sequences | Annealing temp | Productes | cycles |
| --- | --- | --- | --- | --- |
| Oct-4 | 5′-CGACCATCTGCCGCTTTGAG-3′ | 60℃ | 577bp | 30 |
|  | 5′-CCCCCTGTCCCCCATTCCTA-3′ |  |  |  |
| Nanog | 5′-CTGTGATTTGTGGGCCTGAA-3′ | 62℃ | 152bp | 30 |
|  | 5′-TGTTTGCCTTTGGGACTGGT-3′ |  |  |  |
| Sox2 | 5′-CCCCCGGCGGCAATAGCA-3′ | 58℃ | 448bp | 30 |
|  | 5′-TCGGCGCCGGGGAGATACAT-3′ |  |  |  |
| Rex-1 | 5′-GCGTACGCAAATTAAAGTCCAGA-3′ | 56℃ | 350bp | 30 |
|  | 5′-CAGCATCCTAAACAGCTCGCAGAAT-3′ |  |  |  |
| H19 | 5’-AGCTAGAGGAACCAGACCTCATCA-3’ | 62℃ | 525bp | 30 |
|  | 5’-ATGGAATGCTTGAAGGTTGCCC -3’ |  |  |  |
| UBE3A | 5’-ATGCACTTGTCCGGCTAGAGATGA -3’ | 60℃ | 263bp | 30 |
|  | 5’-AGCCAGACCCAGTACTATGCCAAT -3’ |  |  |  |
| PEG3 | 5’-TCGCTGAGGACAGGAAACCT -3’ | 56℃ | 303bp | 30 |
|  | 5’-ACTCCCTTGCTCTTCCCGAT -3’ |  |  |  |
| SGCE | 5’-TACCCATCAGCAGGTGTCCTCTTT -3’ | 60℃ | 265bp | 30 |
|  | 5’-AGGTGCGCCTATTGTAGGCAGTTA -3’ |  |  |  |
| PEG10 | 5’- ACAACAACAACTCCAAGCACACCG -3’ | 60℃ | 322bp | 30 |
|  | 5’- TCTGGGTTGCCATCGAACTTCTCT -3’ |  |  |  |
| ZIM2 | 5’-TGTACCAACCGGAAGACGACAACA -3’ | 60℃ | 228bp | 30 |
|  | 5’-TCGCCATCACAGGAAGGGAAAGAT -3’ |  |  |  |
| MEST | 5’-ATGGGATAACGCGGCCATGGTG -3’ | 56℃ | 218bp | 30 |
|  | 5’-TTCCAACCACACCCACAGAGTCTT -3’ |  |  |  |
| SNPRN | 5’-AGTTACTGTGGATGAGGGTGATGC -3’ | 58℃ | 474bp | 30 |
|  | 5’-CACCCAGGACCTTCCACTCATTTA -3’ |  |  |  |
| NDN | 5’-GAGCCGCCCGAATACGAGTT -3’ | 60℃ | 564bp | 30 |
|  | 5’-GCAGGAGCAGTCTACCCCAA -3’ |  |  |  |
| MAGEL2 | 5’-TAAAGAGCGCAGGACCTCCTCAAA -3’ | 60℃ | 346bp | 30 |
|  | 5’-TGCCTTTGAGGCATTCATGTTGGG -3’ |  |  |  |
| XIST | 5’-TAATGTGCCAGATACCATGCTGGG-3’ | 56℃ | 318bp | 25 |
|  | 5’-ACTTAACCTCACCAGTAAAGTCTTGAT-3’ |  |  |  |
| GAPDH | 5’- TCCACTGGTGTCTTCACGACC -3’ | 56℃ | 120bp | 25 |
|  | 5’- CATGGTTCATGCCCATCACA -3’ |  |  |  |
