# Supplementary material for: Derivation of Rhesus Monkey Parthenogenetic Embryonic Stem Cells and Its MicroRNA Signature
Source: PLoS One. 2011 Sep 26;6(9):e25052. doi: 10.1371/journal.pone.0025052 (PMC3180378; doi:10.1371/journal.pone.0025052)
Supplement: Table S3 — The expression of members of miR-371–373 and C19MC cluster in parthenogenetic rESCs. (DOC) [file pone.0025052.s004.doc]

Supplemental table 3: the expression of members of miR-371–373 and C19MC cluster in parthenogenetic rESCs

| miR | Pa2.2 TPM | Pa3 TPM |
| --- | --- | --- |
| mml-mir-371 | 22.501 | 13.078 |
| mml-mir-372 | 89.736 | 40.943 |
| mml-mir-373 | 296.441 | 141.206 |
| mml-mir-512 | 5.268 | 4.274 |
| mml-mir-498 | 0.000 | 0.085 |
| mml-mir-519c | 0.089 | 0.171 |
| mml-mir-520a | 0.089 | 0.256 |
| mml-mir-526b | 0.089 | 0.000 |
| mml-mir-525 | 0.000 | 0.000 |
| mml-mir-523a | 0.089 | 0.085 |
| mml-mir-518f | 0.089 | 0.256 |
| mml-mir-519a | 0.179 | 0.000 |
| mml-mir-518b | 0.089 | 0.342 |
| mml-mir-518c | 0.179 | 0.256 |
| mml-mir-524 | 0.536 | 0.256 |
| mml-mir-517 | 0.268 | 0.000 |
| mml-mir-519d | 0.000 | 0.000 |
| mml-mir-518a | 0.268 | 0.085 |
| mml-mir-520g | 0.000 | 0.000 |
